# How Do Pharmacists Practice in Aged Care? A Narrative Review of Models from Australia, England, and the United States of America

**DOI:** 10.3390/ijerph182312773

**Published:** 2021-12-03

**Authors:** Ibrahim Haider, Mark Naunton, Rachel Davey, Gregory M. Peterson, Wasim Baqir, Sam Kosari

**Affiliations:** 1 Discipline of Pharmacy, Faculty of Health, University of Canberra, Bruce, ACT 2617, Australia; mark.naunton@canberra.edu.au (M.N.); g.peterson@utas.edu.au (G.M.P.); sam.kosari@canberra.edu.au (S.K.); 2 Health Research Institute, Faculty of Health, University of Canberra, Bruce, ACT 2617, Australia; rachel.davey@canberra.edu.au; 3 School of Pharmacy and Pharmacology, University of Tasmania, Hobart, TAS 7001, Australia; 4 NHS England and NHS Improvement, London SE1 6LH, UK; wasim.baqir@nhs.net

**Keywords:** pharmacists, models of practice, medication review, medication management, residential aged care facilities, long-term care, nursing homes, England, Australia, USA

## Abstract

Medication management in residential aged care facilities (RACFs) is complex and often sub-optimal. Pharmacist practice models and services have emerged internationally to address medication-related issues in RACFs. This narrative review aimed to explore pharmacist practice models in aged care in Australia, England and the USA, and identify key activities and characteristics within each model. A search strategy using key terms was performed in peer-reviewed databases, as well as the grey literature. Additionally, experts from the selected countries were consulted to obtain further information about the practice models in their respective countries. Thirty-six documents met the inclusion criteria and were included in the review. Four major pharmacist practice models were identified and formed the focus of the review: (1) the NHS’s Medicine Optimisation in Care Homes (MOCH) program from England; (2) the Australian model utilising visiting accredited pharmacists; (3) the Centers for Medicare and Medicaid (CMS) pharmacy services in long-term care from the USA; and (4) the Medication Therapy Management (MTM) program from the USA. Medication reviews were key activities in all models, but each had distinct characteristics in relation to the comprehensiveness, who is eligible, and how frequently residents receive medication review activity. There was heterogeneity in the types of facility-level activities offered by pharmacists, and further research is needed to determine the effectiveness of these activities in improving quality use of medicines in the aged care setting. This review found that in some models, pharmacists have a limited level of collaboration with other healthcare professionals, emphasising the need to trial innovative models with integrated services and increased collaboration to achieve a holistic patient-centred approach to medication management.

## 1. Introduction

Medication management in residential aged care facilities (RACFs) is complex and requires the involvement of residents and their families with the multidisciplinary health care team including doctors, nurses, carers, and pharmacists. The role of pharmacists in RACFs is evolving. A rising aging population and increased health care costs in developed Western countries is creating a need for pharmacists to provide solutions to optimise the safety and efficacy of increasingly complex medication regimens in aged care residents. The term RACF is synonymous with “long-term care home” and “nursing home” in different countries.

Residents in RACFs are often prescribed multiple medications and have a high prevalence of medication-related problems (MRPs); over 95% of all residents have at least one MRP [1,2]. Pharmacists are in a unique position to enhance medication management practices in the residential aged care setting. Several systematic reviews have examined the effects of pharmacist-led interventions in RACFs; those interventions, including medication reviews, educational programs, and participation in multidisciplinary meetings, have demonstrated promising results [3,4,5,6,7]. In particular, medication reviews have shown success in identifying potentially inappropriately prescribed medications and have been helpful in improving prescribing practices in the elderly [3,4].

Traditionally, the role of pharmacists in RACFs has focused on delivering pharmacy-related services from a contracted off-site provider to dispense residents’ medications and provide limited services to the facility. More recently, practice models have been evolving where pharmacists have more involvement in residents’ care with expanded clinical activities to improve medication management. For example, in the USA, clinical pharmacy services in RACFs were introduced in the 1960s and have been evolving ever since, with existing pharmacist services accessible on the federal level by the Centers for Medicare and Medicaid (CMS), such as the “Drug Regimen Review” (DRR) and the “Medication Therapy Management” (MTM) services [8,9]. In England, the NHS Medicine Optimisation in Care Homes (MOCH) program was established to incorporate pharmacists into RACFs to conduct on-site medication reviews, as well as other facility-level activities, such as antimicrobial stewardship and collaborating with general practitioners (GPs) to improve residents’ medication regimens. The program was initiated after new models of care were trialled in residential care home settings as part of the NHS Forward Five-Year plan [10].

Pharmacist “practice models” in this review refer to real-world government-funded programs and services that provide clinical services by pharmacists in RACFs. Such practice models are usually well-established and widely adopted within the country [8,9]. To date, there has not been a review that explores different practice models for pharmacists in RACF settings. Recent reviews have examined studies on pharmacist-led interventions, which usually are only trialled for the duration of their respective studies and are typically not widely adopted [3,4,5,6,7]. A recent systematic review and meta-analysis of pharmacist-led interventions and services in RACFs reported on pharmacists’ substantial contribution to patient care in RACFs, resulting in reduced rates of falls [3]. In this systematic review, around 77% of the included studies were from the USA (48%), the UK (10%), and Australia (19%) [3]. Consequently, this review set out to explore the current pharmacist practice models in RACFs in these selected countries (England, Australia, and the USA) to provide an overview to researchers and policy makers on how pharmacists are practicing in RACF settings across different healthcare contexts. The objective was to explore real-world pharmacists’ practice models or services in RACFs, and to identify key activities and characteristics of each model.

## 2. Materials and Methods

This narrative review followed a methodology developed by Cooper and Baumeister [11,12] that used a taxonomy for literature reviews, with six characteristics to define the objectives of the review: (1) focus of attention; (2) goal of the synthesis; (3) perspective on the literature; (4) coverage of the literature; (5) organisation of the perspective; and (6) intended audience. These characteristics were applied to this review to provide the framework described in Table 1.

This review included four major government-funded pharmacist practice models in aged care: one each from England and Australia, and two models from the USA. The practice models were identified through searches of official government agencies and peak national pharmaceutical bodies’ websites of those countries. The list of websites used is available in Appendix A. Experts known to the authors from each selected country were consulted to provide more contextual information about the main national government-funded pharmacist practice models currently in place.

The practice models included in this review are:

- The Australian pharmacist practice model in residential aged care, incorporating Residential Medication Management Review (RMMRs) and quality use of medicines (QUM) services (Australia);
- The Medicine Optimisation in Care Homes (MOCH) program (England);
- The Centers for Medicare and Medicaid (CMS) pharmacy services in long-term care (USA);
- Medication Therapy Management (MTM) (USA).

For each identified practice model, a methodical search of the literature, including grey literature, was conducted to find key relevant documents as outlined in the following section.

### 2.1. Search Strategy: Peer-Reviewed and Grey Literature

A search was conducted of peer-reviewed databases for terms describing the selected models and clinical services. Electronic searches were performed in PubMed and EMBASE using the search keywords presented in Appendix B. The years selected for all searches were from 1980 until August 2021. Titles, abstracts and keywords were screened, and studies that met eligibility criteria were included. Details of documents meeting the eligibility criteria were extracted into a Microsoft Excel file, specifically pertaining to the country, year published, author, pharmacist activities and name of model mentioned in the document.

Due to the nature of the research question, this study included grey literature. This review refers to any publication that is not peer-reviewed as grey literature [13]. To methodically search the grey literature, the study adopted methods used by Godin et al. as a guide [13]. The first search strategy relied on the Google search engine to identify websites relevant to the search terms. Iterations of this search included searching these terms as a phrase, as well as searching “all of these words” in the title of the page. The first 10 pages of the Google result list from each search query were assessed for inclusion by screening titles. Relevant sources were then screened for their executive summaries and table of contents. A separate search was conducted for each of the selected countries. An advanced search was also utilised to increase the likelihood of including relevant results, including adding specific suffixes such as .gov, .gov.au, .edu.au, and .uk. The first author also examined targeted official governmental sources for documents and reports, along with consulting content experts to recommend websites and articles that they felt could be useful to include in the review. The details of the grey literature documents and web pages were manually entered into an Excel file. The information included in the data extraction was the source organisation, title, date published, URL and name of service or model (refer to Appendix B for the full list).

### 2.2. Inclusion Criteria

Documents considered for inclusion were those that contained relevant information on the selected government-funded pharmacist models of practice as outlined above. Only the most recent versions of the document and those available in electronic format and published in English were considered. The review excluded research studies on pharmacist-led interventions that have not been widely adopted in the health care system. Non-clinical pharmacy services, such as dispensing and supply of medications, were not relevant to the objective of this review and, therefore, were excluded. The first and last authors met to discuss the inclusion and exclusion criteria to ensure a common understanding and interpretation of the criteria. In case of disagreement, the third researcher was consulted.

### 2.3. Data Collection Process and Synthesis of Results

The methodology used in this study applied systematic review techniques to the peer-reviewed and grey literature, adhering to the Preferred Reporting Items for Systematic Reviews and Meta-Analyses (PRISMA) to present the data collection process [14]. A narrative synthesis of the literature was conducted inductively according to the focus area described in Table 1.

## 3. Results

**Figure 1:**
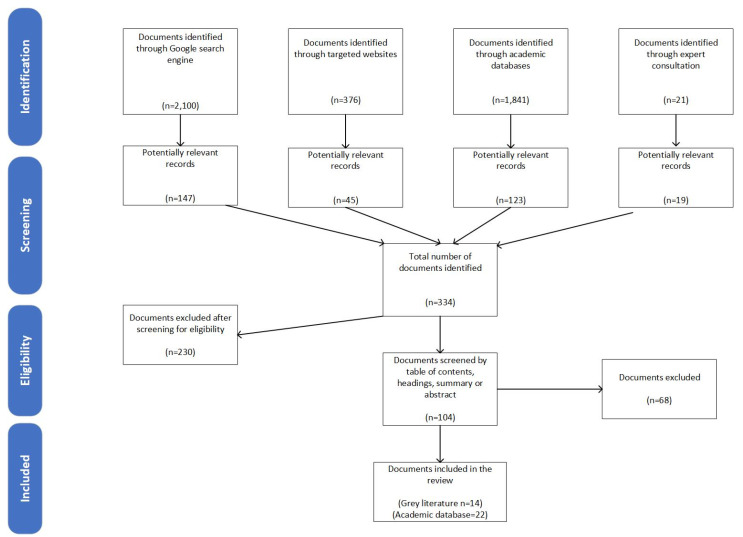
PRISMA flow diagram of document included in this review.

The search strategies retrieved 4338 documents. After screening titles and abstracts for relevance and eligibility criteria, 104 documents/articles were selected for full-text review by the first and second author. Thirty-six documents met the inclusion criteria, which included 22 research articles and 14 documents from grey literature searches. The latter comprised government reports and official guides to selected programs. Figure 1 illustrates the document selection flowchart. 

### 3.1. Characteristics and Activities of Models of Practice

The characteristics and activities of each practice model are summarised in Table 2, Table 3 and Table 4. Table 2 synthesises key characteristics of the models of practice and includes: (a) stated aims of the model, (b) main funding arrangement, (c) type of employment offered, and (d) type of pharmacist qualification/accreditation required. Table 3 summarises medication review activities of the models and describes their eligibility criteria, frequency, and other main attributes. Table 4 lists facility-level activities included in each of the pharmacist practice models. Facility-level activities refer to activities that are not individualised medication review activities (e.g., providing facility-wide education, implementing policies and procedures, antimicrobial stewardship and/or conducting audits).

### 3.2. Australian Pharmacist Model in Residential Aged Care—Residential Medication Management Review (RMMR) and Quality Use of Medicine (QUM) Programs

The Australian model included in this review involves two government-funded pharmacist services—the residential medication management review (RMMR) and quality use of medicines (QUM) services. The RMMR program is a collaborative medication review program, which was started in 1997, allowing an accredited pharmacist to conduct a medication review service for residents in RACFs [18]. The purpose of the RMMR service is to identify and resolve MRPs to improve and achieve positive health outcomes for residents. Upon completion of the medication review, pharmacists send a report with recommendations to the resident’s medical practitioner. Medical practitioners review the RMMR report and act on pharmacists’ recommendations at their discretion. [16,19]. The QUM service aims to improve medication-related practices at a facility-wide level, including conducting activities such as staff training and education, continuous improvement activities, and participation in medication advisory committees. These activities are usually set up as an agreement between a QUM service provider and an RACF. The facility must be provided with a minimum of one QUM activity each quarter to receive the QUM payment [17,21].

Australian studies examining RMMRs have shown promising results in improving QUM indicators for RACF residents. A recent systematic review included eight studies on RMMR interventions, showing effectiveness in identifying MRPs; on average, pharmacists identified 2.7–3.9 MRP per RMMR [4]. The most common MRP type was found to be “undertreatment of conditions”, followed by “medication selection problems” [22].

The most prevalent types of recommendations made by pharmacists after conducting RMMRs were laboratory monitoring followed by dose or schedule changes [4]. The acceptance rate of recommendations by physicians ranged between 45% and 84%, with recommendations related to education and counselling most likely to be accepted [2].

A study examining the impact of RMMRs on reducing the drug burden index (DBI)^®^ found a reduction in DBI score^®^ by half with a notable reduction in benzodiazepine use, as well as the doses of antipsychotic medications [23]. A retrospective study examining the effect of RMMRs on anticholinergic burden using seven different scales found a significant reduction in anticholinergic burden after pharmacists’ recommendations. Additionally, the reduction persisted after accounting for doctors’ acceptance of recommendations [24]. A study examining the effect of RMMRs on regimen complexity using the Medication Regimen Complexity Index (MRCI)^®^ failed to show a significant effect [25].

The effect of RMMRs on specific areas of therapy was examined in three studies [1,26,27]. The appropriateness of prescribing renally cleared medications showed improvements, resulting in 93% GP acceptance rate of recommendations related to the monitoring of renal function [1]. Another cohort study examined the use of antithrombotic medications in residents with atrial fibrillation but found no impact of RMMRs on the prevalence of appropriate use of antithrombotic medications [26]. A study found pharmacists rarely intervened when residents were on QT-prolonging medications, with only 9% of pharmacists intervening when residents were considered at high risk of QT prolongation [27]. The timeliness of conducting RMMRs has been assessed in a study, which showed that only 21.5% of RMMRs were conducted within three months, suggesting a need to intervene earlier [28]. The longer-term impact of RMMRs on resident-specific clinical outcomes is still largely unknown. Additionally, most studies in the literature are retrospective studies with small sample sizes, which may limit interpretation and generalisability [4].

### 3.3. The Medicine Optimisation in Care Homes (MOCH) Program (England)

The Medicines Optimisation in Care Homes (MOCH) program was a result of the Five-Year Forward plan published by National Health Service (NHS) England in 2014, with the aim of transforming the future of health services in England [29].

The MOCH program aims to integrate clinical pharmacists and pharmacy technicians into RACFs for at least 0.4 full-time equivalent [10]. The emphasis of the program is to provide a holistic approach to medication management and optimisation with shared decision making with residents and staff. The clinical services offered within the MOCH program are flexible and can be tailored to meet local needs but must include a direct patient-facing activity such as structured medication reviews, end of life support, or frailty reviews. There is also a focus on supporting frailty through multidisciplinary team meetings (Table 4). In England, the publication of the “Long Term Plan” and the development of clusters of general medical practices known as primary care networks have ensured that the NHS MOCH model of holistic structured medication reviews is now sustained [29].

New care models (also known as vanguard sites) were trialled prior to initiating the NHS MOCH program, and showed promising results with improvements in medication costs and time efficiencies [30]. One example is the Northumberland new care model, which incorporated pharmacists and technicians as part of an enhanced care team in RACFs to improve health outcomes and reduce costs [31]. Over 15 months, the pharmacy team conducted 5124 interventions, with an estimated 223 hospital admissions avoided. Its medicine optimisation interventions contributed to a reduction in polypharmacy from an average of nine medications per resident to seven, with 17.4% of medications ceased [31]. Preliminary evidence of two other vanguard sites that incorporated pharmacists into their care team showed a reduction in the number of hospital admissions [32,33], while the third vanguard study showed no change in hospital utilisation [34]. The Wakefield Enhanced Health in Care Homes had 27% fewer potentially avoidable admissions when compared to a control group [32]. The Nottingham City Clinical Commissioning Group, another vanguard site, had 18% fewer emergency admissions and 27% fewer potentially avoidable admissions in comparison with the control group [33]. The Sutton Homes of Care Vanguard study also reported results on hospital utilisation but showed no change when comparing intervention and control groups [34]. Both the Wakefield Enhanced Health in Care Homes and the Sutton Homes of Care vanguard sites involved a pharmacist as well as other health professionals as part of a broader model of care. This makes it difficult to isolate the effect of pharmacists from the activities conducted by other health professionals.

### 3.4. Centers for Medicare and Medicaid (CMS) Pharmacy Services in Long-Term Care (USA)

The US federal government approved regulations in 1974 assigning pharmacists to oversee and evaluate the drug regimen of each resident in RACFs [35]. Currently, RACFs are required by federal law to obtain consultant pharmacists to oversee several pharmacy services, as set by the Centers for Medicare and Medicaid (CMS). The process is guided by federal tag numbers (or F-tags), which are minimum requirements that must be met to avoid noncompliance [20,36]. The aim of the F-tags is to help facilities meet an acceptable standard of care, and failure to comply results in deficiency citations issued to facilities depending on the severity and impact of deficiency. Consultant pharmacists are needed to supervise pharmacy services, including the conduct of drug regimen reviews (DRRs) for each resident, setting up a system of records for controlled medicines, and developing and reviewing procedures, as well as ensuring adequate storage and labelling standards [9].

The DRR is a medication review that involves reviewing residents’ medication charts to identify and report any drug-related problems. A goal of DRR is to ensure the drug regimen is free from unnecessary drugs [9]. An emphasis has been placed on removing or reducing unnecessary psychotropic medication; to comply with the latest DRR compliance rules, all “when required” (PRN) psychotropic medications should be limited to 14 days of use unless the rationale is clearly stated by the prescriber [37]. The DRR must be conducted once monthly to ensure residents’ medication regimens are free from unnecessary medications. Other pharmacy services that must be overseen by the consultant pharmacists include ensuring low medication error rates and establishing a system of records of receipt and disposition of all controlled drugs. Pharmacists must also establish precise labelling of medications and biologicals to ensure the display of appropriate instructions, cautions and expiration dates. Consultant pharmacists must also oversee adequate storage of medications to comply with state and federal laws [9].

While the DRR service conducted by consultant pharmacists can be effective in reducing the use of psychotropic medications in nursing homes [38], evidence has also pointed out the limited success of the DRR service to improve inappropriate prescribing and reduce the under-treatment of conditions [39]. Proposals to improve the current CMS pharmacy services in RACFs have recommended a more proactive pharmacist role to enhance patient care [40]. A study in the United States evaluated how well the traditional DRR service adheres to clinical practice guidelines when compared to adding a disease state management component for residents after receiving traditional DRR, and the study found a higher rate of adherence to clinical practice guidelines in four out of seven chronic conditions after receiving the added disease state management consultations [41]. Additionally, the Fleetwood landmark research project conducted in the state of North Carolina trialled an alternative consultant pharmacists’ model of care to expand on the conventional CMS pharmacy services by incorporating a more prospective review where residents are prioritised at the time of dispensing to receive a DRR, along with direct communication with prescribers. The authors concluded that extending the CMS pharmacy services was feasible but needed changes in reimbursement to consultant pharmacists. However, the project also found no changes in hospitalisation rate when compared to the conventional CMS DRR service [40].

### 3.5. Medication Therapy Management in Long-Term Care (USA)

Medication therapy management (MTM) is a program aimed to improve medication use and optimise medication-related outcomes by pharmacists and other health care professionals. The MTM program is operated by Medicare part D plan sponsors that can target beneficiaries, provided they meet the eligibility criteria. The MTM program can be offered in any setting, including RACFs [42].

The MTM services involve an annual comprehensive medication review (CMR) and a quarterly targeted medication review (TMR) with follow-up interventions aimed at both prescribers and patients. The comprehensive medication review (CMR) is a systematic and thorough process to review patients’ medication therapy to identify MRPs and create a plan to resolve them with the patient or prescriber. The medication review is conducted in real time with patients or their caregivers, either in person or by telehealth. The targeted medication review (TMR) is initiated on enrolment and aims to assess specific targeted MRPs and monitor any unresolved issues in medication regimens to be assessed [8]. The MTM provider must assess eligibility criteria and coordinate recommendations with the healthcare team members, such as prescribers and consultant pharmacists at the facility. Eligibility criteria include residents who have multiple chronic conditions, take multiple medications covered under Medicare part D plans, and have annual medication costs that are likely to incur a threshold cost.

Research into the effect of MTM services in the US has shown some success in identifying and reducing potentially inappropriate medication use [43,44,45]. In a retrospective study of 9059 Medicare beneficiaries, MTM interventions provided by a clinical pharmacist were associated with a reduction in drugs to avoid in the elderly [44]. The MTM program was evaluated over a period of 10 years in one of the largest healthcare providers in the state of Minnesota; the study showed positive results in relation to decreased drug therapy problems and substantial cost savings of USD 1.29 per USD 1 in MTM administrative costs [45]. There is insufficient evidence, however, for the effect of MTM services on residents’ health outcomes. A systematic review of MTM interventions in outpatient settings concluded that there was inadequate evidence available with respect to improvement in health outcomes, such as disease-specific morbidity, disease-specific or all-cause mortality, and harms. This is mostly due to inconsistency in evidence and heterogeneity in interventions in the populations studied [43].

## 4. Discussion

This article explored four current pharmacist practice models in the RACF setting: the Medicine Optimisation in Care Homes (MOCH) program from England, the Australian pharmacist model in residential aged care (the RMMR and QUM services), and the Centers for Medicare and Medicaid (CMS) pharmacy services in long-term care and medication therapy management (MTM) from the United States. The review aimed to provide an overview, synthesise the available evidence, and identify key clinical and non-dispensing activities conducted by pharmacists for each of the selected pharmacist practice models.

### 4.1. Medication Review Activities

Medication review activities constitute the key component of the four presented models. There are important differences in how pharmacists conduct medication reviews in each of the models. The differences mainly relate to eligibility criteria, frequency of medication reviews recommended per resident, and the comprehensiveness of the medication review activity. The MOCH model in England integrates a flexible structure where pharmacists and facilities can decide the type and frequency of medication review activities that best suit the needs of residents. The program structure allows pharmacists to establish a risk stratification strategy to select which residents are prioritised according to the local needs of each facility [10]. In contrast, the Australian model recommends accredited pharmacists to conduct RMMRs usually every 24 months, in addition to two follow-ups within 9 months post RMMR, or occasionally more frequently if a resident is at risk of medication misadventure or has been recently discharged from hospital, according to specific eligibility criteria [18,19]. Conducting RMMRs reactively after a resident has experienced a recent health event has been criticised by medical practitioners [46,47]. Instead, a more proactive approach to conduct a medication review may help prevent medication-related adverse outcomes before they occur. In the United States’ CMS model, consultant pharmacists are required to conduct drug regimen reviews (DRR) for all residents on a monthly basis for the purpose of ensuring there are no unnecessary medications [9]. However, the DRR is a medication chart review that lacks the comprehensiveness and systematic approach of other models. On the other hand, the United States’ MTM program involves a comprehensive annual medication review along with more targeted reviews conducted quarterly [8]. However, a drawback for the MTM program is it is limited to residents who are only covered by the Medicare Part D insurance plans and meet its stringent eligibility criteria [8].

### 4.2. Facility-Level Activities

Pharmacists’ involvement in facility-level activities varies considerably in the presented models; for example, England’s MOCH program’s commitment to antimicrobial stewardship and the use of data and technology to support medicine optimisation [10]. The Australian model offers quality use of medicines (QUM) services to improve medicines-related practices in RACFs, which include participating in medication advisory committees, education, and continuous quality improvement activities [18], although with limited evidence of the effectiveness [48]. Pharmacists working within the CMS model in the USA are consulted on procedures and policies related to the provision of medication services in the facility. Those activities include establishing a system of records for controlled drugs, ensuring adequate storage, and labelling of drugs and biologicals [9]. The MTM program in the US is an exception in that it only focuses on providing comprehensive medication reviews (CMRs) and targeted medication reviews specific to residents, and does not support any facility-level services [8].

There is variation in facility-level activities provided by the practice models presented. As medication experts, pharmacists’ participation in facility-level activities, such as providing education and contributing to policies, may help improve the quality of medication management practices in RACFs. However, evidence on the effectiveness of such activities is still limited. A systematic review by Lee et al. found that pharmacist interventions such as education improved the knowledge of health care workers but was unable to generalise findings due to the poor quality of studies and limited sample sizes [3]. A qualitative study found healthcare professionals often recommended conducting facility-level audits and feedback to staff as a potential solution to reduce polypharmacy [49]. Further evidence is needed on which facility-level activities should be routinely adopted by pharmacists in RACF settings.

### 4.3. Current Evidence for the Practice Models

The evidence for the Australian RMMR model, as well as both models from the USA, has shown effectiveness in identifying MRPs [4,38,45]. In fewer studies, those models have also shown success in reducing MRPs [39,44,50]. The MOCH program in England has shown improvements in medication costs and time efficiencies from smaller scale pilot studies, which have not yet been published in peer-reviewed literature [32,33]. For the most part, the evidence for the pharmacist models of practice explored in this review, as well as other pharmacist-led interventions in RACFs, has focused on the effectiveness in identifying and reducing MRPs. Further research is needed to identify the effects of pharmacists’ models on resident-specific outcomes, such as hospitalisations, mortality rate and quality of life [51]. While this review did not assess the quality of evidence presented, two systematic reviews of pharmacist interventions in aged care have indicated that many studies are of lower quality designs, such as pre- and post-studies, and have small sample sizes [3,4]. Therefore, there is a need for better quality studies, such as randomised controlled trials with large sample sizes, to determine the impact of the presented models.

### 4.4. Level of Collaboration

Pharmacists’ level of collaboration with the multidisciplinary healthcare team differs amongst the selected models. In England, the MOCH program places an emphasis on integrating pharmacists within the multidisciplinary team and highlights the importance of engagement with medical practitioners. The program also recommends the participation of residents and their families in the decision-making processes [10]. In contrast, the Australian model, as well as the two US pharmacist models presented in this review, place less emphasis on integrating pharmacists as part of the multidisciplinary healthcare teams in RACFs. In particular, there is a lack of information on pharmacists’ interaction with nurses when conducting medication review activities in aged care. It is sometimes mentioned as part of the multidisciplinary team in the MOCH program [10,15]. Medication review activities generally involve interaction with residents and then recommendations are communicated and considered by medical practitioners. A pilot study in Australia trialled embedding a pharmacist to conduct integrated services as part of the RACF healthcare team [52]. The study suggested that performing integrated services by a pharmacist collaboratively within the RACF healthcare team increased the participation of stakeholders during medication reviews and helped the delivery of a more holistic patient-centred service to residents [53]. Increasing the collaboration of pharmacists with the RACF’s multidisciplinary team may help improve the current practice models and increase pharmacists’ and residents’ involvement in decision making [54].

### 4.5. Future Directions

The role of pharmacists in the residential aged care setting is evolving. Pharmacists are increasingly expanding their range of activities performed in RACFs. However, high-quality studies examining integrated pharmacist services in the aged care setting are still needed to support and develop this role in the future. Currently, there are two cluster randomised controlled trials underway in the UK and Australia, which are exploring novel practice models in aged care where pharmacists are working collaboratively with the multidisciplinary team. The Care Home Independent Prescribing Pharmacist Study (CHIPPS study) in the UK is a five-year research programme into medicines management in care homes, aiming to determine the clinical benefits and cost-effectiveness of a pharmacist-independent prescribing service in RACFs compared to usual GP-led care; the intervention involves conducting medication reviews, prescribing, training and support, and communication [55]. In Australia, the pharmacist in residential aged care facilities study (the PiRACF study) aims to evaluate the effectiveness of an integrated pharmacist practice model within a multidisciplinary team in the aged care facility [56]. This new model involves embedding a pharmacist as part of the multidisciplinary health care team for two days a week to conduct medication management activities alongside nurses, carers and doctors. Medication management activities include conducting medication reviews, clinical audits, education and being involved with facility policies and procedures. If such studies are successful and show cost-effectiveness, they may have implications for future polices as governments may be more likely to fund integrating pharmacists in residential aged care settings as part of multidisciplinary healthcare teams. This evolving role in the future also has implications for pharmacy education as pharmacists must enhance their collaboration and communication skills to successfully adopt a more multidisciplinary approach to medication use and safety [57].

### 4.6. Limitations

There are limitations to the findings reported in this review. The review did not aim to include all pharmacists’ activities related to medication management in RACFs in the countries selected. The findings were synthesised from selected documents and peer-reviewed articles. Therefore, the application of this in practice may somewhat differ. Nonetheless, the authors took considerable effort in trying to ascertain all information found with discussions with pharmacists from selected countries.

## 5. Conclusions

This review presented four real-world pharmacist practice models in the residential aged care setting and examined key activities and characteristics within each model. Medication review was the cornerstone activity of all four models, each with different attributes regarding eligibility criteria, frequency, and level of comprehensiveness. Conversely, there was greater heterogeneity in the types of facility-level activities conducted by pharmacists amongst the different models. Further research is needed to determine which facility-level activities are most effective in the aged care setting. In some models, pharmacists’ activities indicated a limited level of collaboration with the rest of the healthcare team in the facility, emphasising a need to trial innovative models with integrated services and increased collaboration for a holistic patient-centred approach to medication management.

## Figures and Tables

**Table 1:** Taxonomy of this review according to Cooper [11].

**Focus**	Research outcomes	Research articles and documents on pharmacist practice models offering clinical services in aged care facilities within selected countries.
**Goal**	Identification of central issues, integration/generalisation	(i) Identify and describe international pharmacist models of practice in aged care in selected countries (England, Australia and the USA), (ii) synthesise documents to characterise each practice model based on resident-level and facility-level activities, employment type and pharmacist qualifications, and (iii) provide an overview of the available evidence for benefits.
**Perspective**	Neutral representation	Research findings are presented in an unbiased manner, as in the original documents.
**Coverage**	Representative	Pharmacist practice models will be selected based on selection criteria, and studies and documents are selected to represent the specified models and their evidence. The coverage will not be exhaustive of all relevant models and studies.
**Organisation**	Conceptual	Articles and documents relating to each country’s practice model(s) are represented together.
**Audience**	Health researchers, practitioners, policy makers	Informing stakeholders such as policy makers and researchers on developing international models of pharmacist practice in aged care, the current activities within those models, and their evidence for benefits.

**Table 2:** Key characteristics of the selected pharmacists’ models of practices in RACFs.

	MOCH—England [10,15]	RMMR and QUM Services—Australia [16,17,18,19]	CMS in Long-Term Facilities—USA [9,20]	MTM—USA [8]
Stated aims of the model/service	To train and deploy clinical pharmacists and pharmacy technicians into care home settings to improve quality of care through better medicines use, savings and waste reduction.	To improve the patient’s quality of life and health outcomes using a best practice approach, detect and address medicine-related problems, and provide education to residents, carers and other healthcare providers.	To obtain services of a licensed pharmacist by facilities to ensure the safe and effective use of medications and other pharmaceutical services.	To improve medication use, reduce the risk of adverse events, and improve medication adherence.
Main funding arrangement	Funded fully by NHS England’s Pharmacy Integration Fund in year 1, and subsequently 50% of costs is covered by local commissioning group (Clinical Commissioning Groups, in England).	Funded by the Australian Government Department of Health & Ageing under the 7th Community Pharmacy Agreement.	Facilities funded by the Centers for Medicare and Medicaid (CMS) must meet their requirements, which include obtaining the services from consultant pharmacists to oversee pharmacy services for the long-term care (LTC) facility.	Medicare Part D plan sponsors are funded federally by the Centers for Medicare and Medicaid Services through the Medicare Part D program.
Type of Employment	Pharmacy professionals are employed by a range of employers (including NHS hospitals, GP practices, community hospitals, community pharmacy, commissioning organisations)—all employers were commissioned by clinical commission groups (CCG) to work on a part-time basis depending on model.	Consultant pharmacists work as independent contractors and are compensated per service from the Community Pharmacy Agreement funds.	Consultant pharmacists can be self-employed, employed by the facility, or employed by a pharmacy provider.	Medicare Part D plan sponsors * set contracts and the fee structure to remunerate pharmacists to provide MTM services.
Type of qualification/accreditation required	Licensed pharmacist. Pharmacists participate in an 18-month training pathway, including the UK’s independent pharmacist prescribing pathway.	Licensed pharmacist and additional accreditation with an approved professional body such as the Association of Consultant Pharmacists (AACP) or the Society of Hospital Pharmacists of Australia (SHPA). The accreditation is renewed every 3 years by examination.	Licensed Pharmacist in state/jurisdiction.	Licensed pharmacist in state/jurisdiction.

* Part D plan sponsors are non-governmental organisations under contract with CMS to offer prescription drug benefits and MTM programs.

**Table 3:** Medication review activities.

	MOCH—England [10,15]	RMMR and QUM Services—Australia [16,17,18,19]	CMS in Long-Term Facilities—USA [9,20]	MTM—USA [8]
Description of Activity	The model incorporates direct patient-facing activities within a shared decision-making framework depending on local needs (e.g., structured medication reviews, end of life support, frailty reviews).	Involves a systematic review of resident’s medication regimen.	Drug Regimen Review (DRR) is a review of the medical chart of each resident to report and act on irregularities and must ensure residents are free from unnecessary medications.	Involves a comprehensive review of medications.
Eligibility criteria to receive activity	Activity must contain a risk stratification strategy to prioritise residents in need of medication review.	Residents must meet eligibility criteria (e.g., the patient is at risk of, or currently experiencing, medication misadventure).	All residents must be reviewed.	Eligible Medicare Part D * recipients who meet the eligibility requirements can be targeted by Part D plan sponsors, such as those residents with multiple chronic conditions, multiple Part D covered medications, especially those incurring high annual medication costs.
Frequency of service	As required, no restriction.	Permanent residents in accredited RACFs are eligible to receive an RMMR every 24 months or if deemed clinically necessary by the prescriber, with 2 follow-ups if required	A monthly review by consultant pharmacist	Involves an annual comprehensive medication review (CMRs) and targeted medication reviews (TMRs) at least quarterly with follow-up interventions when necessary.
Communication	Pharmacists must be able to access care home resident/GP records and appropriate data with adequate information technology support. Pharmacists must engage directly with GP Practices responsible for the primary health care of patients.	Where appropriate, the accredited pharmacist and the referring medical practitioner should discuss the findings, recommendations and suggested medicines management strategies, either by phone or face to face.	The pharmacist must document any identified irregularities in a separate written report. The report may be in paper or electronic form. The pharmacist’s findings are considered part of each resident’s medical record. The pharmacist is also responsible for reporting any identified irregularities to the attending physician, the facility’s medical director, and director of nursing.	Plan sponsors are encouraged to adopt standardised health information technology (HIT) for documentation of MTM services. The MTM provider should coordinate the recommendations for drug therapy changes as a result of an MTM encounter with the beneficiary’s treating physician and healthcare team at the facility.
Other attributes	Support arrangements for those with cognitive disabilities and palliative care. A focus on resident and family’s involvement in the decisions-making	An RMMR is initiated by GP referral, pharmacist sends recommendations to resident’s GP, then a medication management plan is developed.	A focus on reviewing psychotropic medications (i.e., PRN orders for psychotropics are limited to 14 days). Facility must ensure medication error rate is less than 5 percent.	Resident’s CMR may be conducted person-to-person, or via a telehealth consultation. Involves a summary with a personalised medication action plan and medication list for the residents. Promote coordinated care, intervention recommendations must target both residents and prescribers.

* Medicare Part D refers to a United States federal-government program to help Medicare beneficiaries pay for self-administered prescription drugs through prescription drug insurance premiums.

**Table 4:** Types/characteristics of facility-level activities included in models of practice.

MOCH—England [10,15]	RMMR and QUM Services—Australia [16,17,18,19]	CMS in Long-Term Facilities—USA [9,20]	MTM—USA [8]
- Support antimicrobial stewardship; - Commitment to supporting frailty through working with a multidisciplinary team; - Integration as part of the multidisciplinary health care team; - Engage and collaborate with GP practices and community pharmacists; - Support care homes and their staff with medicines management tasks (e.g., ordering and safe storage of medicines); - Support nursing staff with medicines administration.	QUM services can include any of the following activities: - Medication advisory activities; - Education activities; - Continuous quality improvement activities.	A licenced pharmacist must be consulted on provision of pharmacy services in the facility, including: - Establishing a system of records of receipt and disposition of all controlled drugs; - Ensure adequate labelling and storage of drugs and biologicals in accordance with State and Federal laws.	Provides resident specific services only and does not provide any facility-level activities.

## Abbreviations

RACF	Residential aged care facility
LTC	long-term care facility
PIM	Potentially inappropriate medication
RMMR	Residential medication management review
GP	General practitioner
QUM	Quality use of medicines
NHS	National health services
MOCH	Medicine optimisation in care homes model
CMS	Centers for Medicare and Medicaid pharmacy services
MTM	Medication therapy management
TMR	Targeted medication review
CMR	Comprehensive medication review

## Appendix A

**Table A1:** List of websites of pharmaceutical peak bodies and governmental organisations used to search for pharmacist practice models.

Number	Government Organisation	URL Link
1	The National Health Service (NHS) England	https://www.england.nhs.uk/ (accessed on 8 February 2021)
2	Royal Pharmaceutical Society of Great Britain	https://www.rpharms.com/ (accessed on 15 October 2020)
3	Centers for Medicare & Medicaid Services	https://www.cms.gov/ (accessed on 23 February 2021)
4	American Pharmacists Association	https://www.pharmacist.com/ (accessed on 17 March 2021)
5	American Society of Consultant Pharmacists (ASCP)	https://www.ascp.com/ accessed on 23 February 2021)
6	Pharmaceutical Society of Australia (PSA)	https://www.psa.org.au/ (accessed on 20 February 2021)
7	Australian Government Department of Health	https://www.health.gov.au/ (accessed on 17 March 2021)
8	Pharmacy Programs Administrator (PPA)	https://www.ppaonline.com.au/ (accessed on 25 February 2021)

## Appendix B

**Table A2:** Keywords and terms used in search strategies.

Search #	Concept	Other Terms
#1	Pharmacist	pharmacist, pharmacists, pharmaceutical service, pharmaceutical services, pharmacy”, “pharmacists
#2	Keywords of selected models	Centers for Medicare and Medicaid pharmacy services, drug regimen reviews, DRRs, Australian model, Residential Medication Management Review, RMMRs, QUMs, MACs, medication therapy management, MTM, medicines optimisation in care homes, vanguards, NHS, MOCH
#3	Residential aged care settings	aged care, aged patient care, aged patient services, assisted living, care home, elder care, elder patient care, long term care, long-term care, nursing home, older person care, older patient care, patient aged care, residential care, residential aged care, skilled nursing facility
#4	Selected countries	England, English, USA, The United States, United States of America, American, Australia, Australian.
